# *Lissonota holcocerica* Sheng sp.n (Hymenoptera: Ichneumonidae) parasitizing *Holcocerus hippophaecolus* (Lepidoptera: Cossidae) from China

**DOI:** 10.1673/031.012.11201

**Published:** 2012-10-05

**Authors:** Shi-Xiang Zong, Mao-Ling Sheng, You-Qing Luo, Chang-Kuan Lu

**Affiliations:** ^1^ The Key Laboratory for Silviculture and Conservation, Ministry of Education, Beijing Forestry University, Beijing, 100083, P.R. China; ^2^ General Station of Forest Pest Management, State Forestry Administration, Shenyang 110034, P.R. China; ^3^ Hebei Normal University of Science and Technology, Qinhuangdao, 066600, P.R. China

**Keywords:** Banchinae, bionomics, parasitoid, taxonomy

## Abstract

This paper describes *Lissonota holcocerica* Sheng sp.n. (Hymenoptera: Ichneumonidae), parasitizing *Holcocerus hippophaecolus* Hua, Chou, Fang and Chen (Lepidoptera: Cossidae) in Inner Mongolia, Liaoning, and Ningxia, China. The adults of *L. holcocerica* emerge from a cocoon during the daytime, mostly from 09:00 to 14:00 hr, then start calling and mating, mostly from 08:00 to 12:00 hr. Mating lasts from 15 sec to 15 min. The longevity of males is 5.8 ± 2.4 days, whereas for females longevity is 7.6 ± 4.6 days in the field. Nutritional supplements can significantly extend the life span of *L. holcocerica*. Mated females search for hosts by walking on the base of *Hippophae rhamnoides* L. (Rosales: Elaeagnaceae) stem infested by *Ho. hippophaecolus*, vibrating their antennae and tapping while searching the stem for oviposition sites. Parasitized cocoons of *Ho. hippophaecolus* collected in field were half the size of normal ones or even smaller.

## Introduction

The sea buckthorn carpenterworm, *Holcocerus hippophaecolus* Hua, Chou, Fang and Chen (Lepidoptera: Cossidae), attacks mainly the roots and trunks of *Hippophae rhamnoides* L. (Rosales: Elaeagnaceae), a commercially important deciduous shrub in the northern regions of China, thus making most of them hollow and eventually leading to the death of the plant. In recent years, *Ho. hippophaecolus* populations have sharply increased in Inner Mongolia and Liaoning, Shanxi, Ningxia, Shaanxi, and Gansu provinces in China, which has aggravated the damage to *Hi. rhamnoides* populations and severely impaired the local eco-environmental construction and economic activities based on this species (Zong et al. 2005, 2011; Luo et al. 2007; Tao et al. 2012). The authors studied the parasitoids of *Ho. hippophaecolus*, and an important species, *Lissonota holcocerica* Sheng sp.n., was found and investigated.

*Lissonota* Gravenhorst, 1829, belonging to subfamily Banchinae (Hymenoptera: lchneumonidae), comprises 388 described species (Yu et al. 2005). The Oriental species were studied by Chandra and Gupta (1977). So far 24 species are known from China (Sheng 2000; Sheng and Li 2006; Sheng and Sun 2009, 2010). The biology was reported by Gauld et al. (2002). The status of the genus was elucidated by Townes (1970) and Townes and Townes (1978).

The holotype and six paratypes are deposited in the Insect Museum, General Station of Forest Pest Management (GSFPM), State Forestry Administration, P.R. China. Two paratypes have been deposited in the Natural History Museum, London, U.K. (BMNH).

## Materials and Methods

Cocoons of *Ho. hippophaecolus* were collected from *Hi. rhamnoides* forest heavily infested by *Ho. hippophaecolus* during the periods of adult emergence, and all cocoons collected were dissected to record the number of parasitic and non-parasitic cocoons. Two square nylon cages (100 × 100 × 100 cm) were set up outdoors, and 10 virgin males and 10 virgin females of *L. holcocerica* were placed in pairs into each cage. One cage only had water while the other cage had both water and 10% honeydew. The feeding, mating, oviposition, and longevity behaviors were then observed. Additional observations were made in the field on *Ho. hippophaecolus* infested forests from which *L. holcocerica* emerged naturally.

The morphological terminology is mostly that of Gauld (1991). Wing vein nomenclature is based on Ross (1936) and the terminology on Mason (1986, 1990).

## Results and Discussion

**Description**
Genus *Lissonota* Gravenhorst, 1829 *Lissonota* Gravenhorst, 1829.
  Ichneumonologia Europaea, 3:30. Typespecies: *Lissonota sulphurifera* Gravenhorst, 1829.
**Diagnosis.** Face wider than long. Apical margin of clypeus thick. Malar space 0.4 to 1.3 times as long as basal width of mandible. Occipital carina complete, lower end joining oral carina some distance above base of mandible. Epomia absent or short and weak. Propodeum usually with posterior transverse carina, rarely absent. Hind wing vein 1-cu slightly longer than cu-a. Tarsal claws usually with distinct pectination. First tergum moderately narrowed toward the base, with a rather abrupt constriction at base. Ovipositor sheath 1.1 to 4.5 times as long as hind tibia.

**Figure 1.**
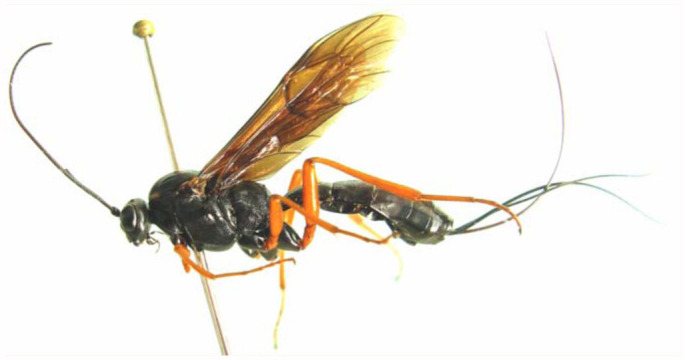
*Lissonota holcocerica* Sheng, sp.n. (I) Body of female, lateral view. High quality figures are available online.

**Figure 2.**
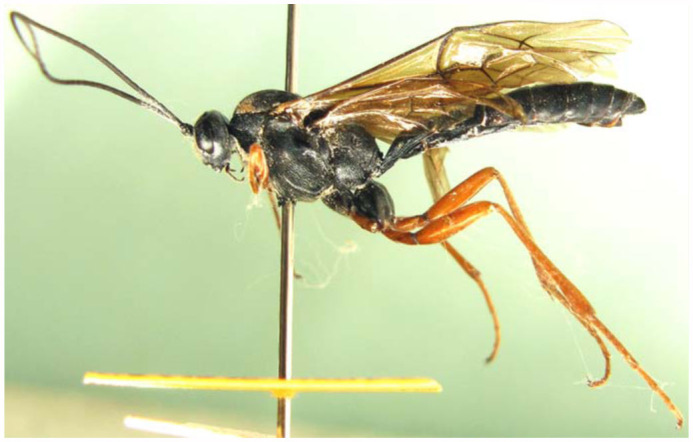
*Lissonota holcocerica* Sheng, sp.n. (2) body of male, lateral view. High quality figures are available online.

**Figure 3.**
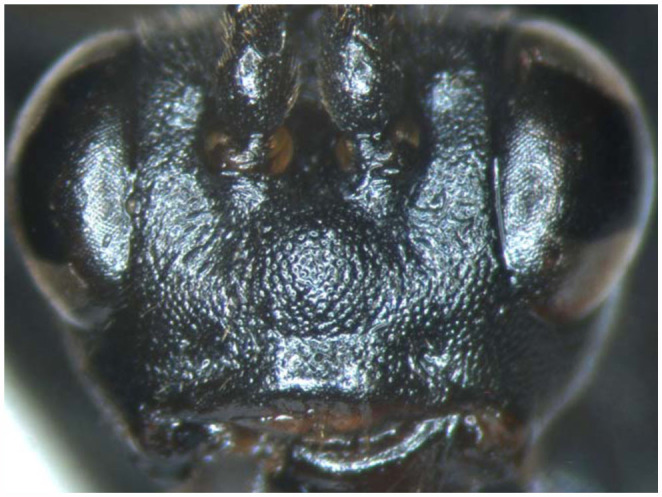
*Lissonota holcocerica* Sheng, sp.n. (3) face. High quality figures are available online.

**Figure 4.**
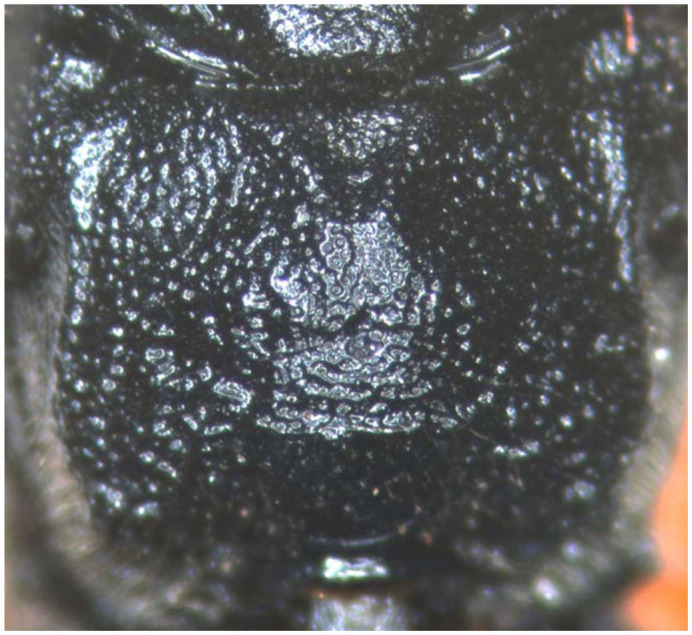
*Lissonota holcocerica* Sheng, sp.n. (4) propodeum. High quality figures are available online.

**Figure 5.**
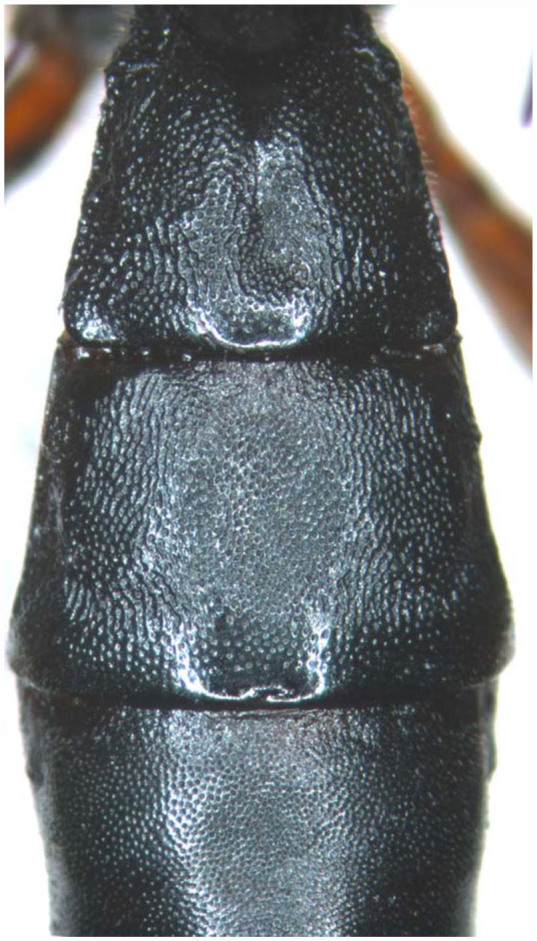
*Lissonota holcocerica* Sheng, sp.n. (5) basal portion of matasoma, dorsal view. High quality figures are available online.

***Lissonota holcocerica* Sheng, sp.n. (Figures 1, 2, 3, 4, 5)**
**Etymology.** The name of the new species is based on the host's name.
**Types.** Holotype, female, CHINA: Jianping County, Liaoning Province, 20 June 2003, leg. Chang-Kuan Lu and Shi-Xiang Zong (GSFPM). Paratypes: 2 females, CHINA: Wulateqanqi, Inner Monggol Autonomous Region, 14 July 1978, leg. He-Ming Chen (GSFPM). 1 female, CHINA: Kazuo County, Liaoning Province, 11 August 1983, leg. Guang Wu (GSFPM). 1 female, CHINA: Huanren County, Liaoning Province, August 1984, leg. Wen-Long Han (GSFPM). 1 female and 1 male, CHINA: Dongsheng, Inner Monggol Autonomous Region, 15 July 2002, leg. Chang-Kuan Lu and Shi-Xiang Zong (BMNH). 2 males, CHINA: Jianping County, Liaoning Province, 20 June 2003, leg. Chang-Kuan Lu and Shi-Xiang Zong (GSFPM). 1 female and 1 male, CHINA: Dongsheng, Inner Monggol Autonomous Region, 6 to 15 June 2006, leg. Mei Su (GSFPM). 1 female, CHINA: Dongsheng, Inner Monggol Autonomous Region, 10 July 2006, leg. Mao-Ling Sheng (GSFPM). 1 female, CHINA: Kuandian County, Liaoning Province, 30 June 2009, leg. Xiao-Yi Wang (GSFPM).
**Diagnosis.** Wing brown. Ratio of length of hind tarsomeres 1:2:3:4:5 is 10.0:4.5:3.0:1.5:2.7. Propodeum irregularly rough, median portion irregularly and strongly convex, basal portion triangularly concave inversely. First and second terga with dense and distinct punctures. Ovipositor sheath 2.5 to 3.0 times as long as hind tibia.
**Description.** Female. Body length 13.5 to 20.0 mm. Fore wing length 11.0 to 16.5 mm. Ovipositor sheath length 16.0 to 19.5 mm.
**Head.** Face (Figure 3) 2.1 to 2.2 times as wide as long, with dense punctures, median portion strongly convex, upper lateral with concavity close to antennal socket. Clypeal suture distinct. Median portion of clypeus strongly convex transversely, basal portion smooth with sparse and indistinct punctures, apical portion smooth and impunctate. Mandible relatively long, with sparse fine punctures; upper tooth approximately as long as lower tooth. Cheek with dense, shallow and indistinct punctures. Malar space approximately as long as basal width of mandible. Gena smooth, with distinct punctures, distance between punctures 1.4 to 3.0 times diameter of puncture; straightly convergent backward; in lateral view 0.6 to 0.7 times as long as width of eye. Vertex with irregular punctures. Postero-ocellar line about 0.9 times as long as ocular-ocellar line. Median portion of firons deeply concave, with smooth median longitudinal groove, upper and lateral portion with dense punctures. Antenna filiform, with 39 to 40 flagellomeres. Ratio of length of flagellomere 1:2:3:4:5 is 10.0:7.0:6.3:6.0:5.8. Occipital carina complete and strong, joining oral carina distinctly above base of mandible.
**Mesosoma.** Anterior portion of pronotum with weak and indistinct punctures; upper portion of lateral concavity with short transverse wrinkles, lower and posterior portions with distinct punctures, distance between punctures 0.3 to 1.5 times diameter of puncture. Mesoscutum with dense punctures, distance between punctures 0.2 to 2.0 (sublateral-median about 2.5) times diameter of puncture. Notaulus indistinct. Scutellum slightly convex, with irregular punctures. Postscutellum slightly convex, anterior-lateral portion concave. Mesopleuron with punctures similar to that of mesoscutum, slightly larger than that; median portion with irregular transverse concavity. Epicnemial carina strong, upper end reaching about 0.3 distance to subalar prominence. Speculum distinct. Metapleuron with dense punctures, distance between punctures 0.5 to 2.5 times diameter of puncture. Juxtacoxal carina absent. Anterior half of submetapleural carina strongly lobed. Wings brown. Fore wing with vein 1cu-a distal of 1-M. Areolet slanting quadrangular, receiving 2m-cu at its middle. Vein 2-Cu approximately 2.0 times as long as 2cu-a. Hind wing vein 1-cu slightly longer than cu-a. Claw small, pectinate. Ratio of length of hind tarsomeres 1:2:3:4:5 is 10.0:4.5:3.0:1.5:2.7. Propodeum (Figure 4) irregularly rough, basal-lateral portion with distinct punctures, median portion irregularly and strongly convex, basal portion triangularly concave inversely, posterior portion behind posterior transverse carina strongly declining. Propodeal spiracle oval.
**Metasoma.** Metasoma robust, apical portion slightly compressed. First tergum about 1.2 times as long as apical width, with dense and distinct punctures, lateral portion rough, subapical with weak and short longitudinal wrinkles, basal-median portion concave and smooth, apical half with irregular longitudinal median groove. Median dorsal carinae vestigial basally. Spiracle located at basal 0.3 of first tergum. Second tergum (Figure 5) 0.7 to 0.8 times as long as apical width, with dense and distinct punctures, distance between punctures 0.2 to 1.0 times diameter of puncture, apical margin smooth narrowly. Third tergum with dense and more finer punctures than that of second tergum, apical margin smooth narrowly. Fourth tergum with distinct punctures, the punctures sparser and finer than that of third. Fifth tergum with very sparse, irregular and fine punctures. Remaining terga smooth. Apical margin of eighth tergum almost truncated. Apex of hypopygium with a triangular notch. Ovipositor sheath approximately 2.5 to 3.0 times as long as hind tibia.
**Color** (Figure 1). Black, except the following. Apical portion of clypeus, legs, except coxae black and hind tarsi dark brown, brown to reddish brown. Spots on lateral-anterior portion of mesoscutum, tegulae and spots of subalar prominences yellow to yellowish brown.
**Male** (Figure 2). Body length 18.5 to 19.5 mm. Fore wing length 14.5 to 15.0 mm. Antenna with 42 flagellomeres. Ventral profiles of second trochanters and femora, apical portions of hind tibiae blackish brown.
**Host.** *Holcocerus hippophaecolus* Hua, Chou, Fang and Chen (Lepidoptera: Cossidae). Endoparasitism.
**Host food.** *Hippophae rhamnoides* L. (Rosales: Elaeagnaceae).

This new species is similar to *L. setosa* (Geoffroy 1785), but can be easily distinguished by the following key.

In Sheng and Sun's key to species (2010), the new species can be inserted as follows:

20. Frons flat or almost flat. Postero-ocellar line as long as or longer than ocular-ocellar line. Ovipositor sheath as long as length of body 21
Frons concave deeply, with a smooth median longitudinal groove. Posteroocellar line shorter than ocular-ocellar line. Ovipositor sheath shorter or longer than length of body 20′
20′. Wing distinctly brown. First to fourth terga with dense and regular punctures *L. holcocerica* Sheng
Wing hyaline, very slightly infuscate. The sculpture on first to fourth terga is quite irregular, tending towards aciculaterugose, with punctures, especially at the apex of the first tergite and on the second *L. setosa* (Geoffroy)

**Bionomics**
The host of *L. holcocerica* is *Ho. hippophaecolus* in living wood of *Hi. rhamnoides*.

Adults emerge during the daytime, mostly from 09:00 to 14:00 hr at room temperature. Newly emerged adults chew a round hole with mouthparts in the epidermis of the cocoons, and then crawl out quickly. Adults groom their antennae and wings after emerging, and then take off, heading towards light places indoors.

Virgin females start their mating behaviors after their adult emergence; mating occurs during the daytime, mostly from 8:00 to 12:00 hr at room temperature, and lasts from 15 sec to 15 min. Before copulation, female wasps chase males, flying around in the rearing cage. After mating, males rest for a while then show gestures of wanting to copulate with other females, but usually they are rejected by females. Copulated females sit still and reject males.

Mated females search for hosts by walking on the base of *Hi. rhamnoides* stem infested by *Ho. hippophaecolus* while vibrating their antennae, tapping the stem while searching for oviposition sites. In field, female wasps were seen walking around at the base of trunks, swinging their antenna to detect larvae. Females laid eggs into bodies of *Ho. hippophaecolus* larvae that lived in the stems of *Hi. rhamnoides*. After hatching, larvae of wasps fed inside the host larvae. Parasitized moth larvae dug into the soil and formed a cocoon earlier than normal. After depleting all the nutrients of the moth larvae, wasp larvae made membranous cocoons and pupated. Parasitized cocoons collected in field were half the size of normal ones or even smaller.

Male longevity was 5.8 ± 2.4 days whereas female longevity was 7.6 ± 4.6 days in the field. Nonetheless, the longevity of males and females was 5.5 ± 1.8 days when only fed with some water and 7.8 ± 4.0 days when fed with some water and 10% of honey dew (syrup). Therefore, the results show that nutritional supplements can significantly extend the life span of *L. holcocerica*.
